# Recurrent Rare Genomic Copy Number Variants and Bicuspid Aortic Valve Are Enriched in Early Onset Thoracic Aortic Aneurysms and Dissections

**DOI:** 10.1371/journal.pone.0153543

**Published:** 2016-04-19

**Authors:** Siddharth Prakash, Shao-Qing Kuang, GenTAC Registry Investigators, Ellen Regalado, Dongchuan Guo, Dianna Milewicz

**Affiliations:** Department of Internal Medicine, Division of Medical Genetics, University of Texas Health Science Center at Houston, Houston, Texas, United States of America; Johns Hopkins University, UNITED STATES

## Abstract

Thoracic Aortic Aneurysms and Dissections (TAAD) are a major cause of death in the United States. The spectrum of TAAD ranges from genetic disorders, such as Marfan syndrome, to sporadic isolated disease of unknown cause. We hypothesized that genomic copy number variants (CNVs) contribute causally to early onset TAAD (ETAAD). We conducted a genome-wide SNP array analysis of ETAAD patients of European descent who were enrolled in the National Registry of Genetically Triggered Thoracic Aortic Aneurysms and Cardiovascular Conditions (GenTAC). Genotyping was performed on the Illumina Omni-Express platform, using PennCNV, Nexus and CNVPartition for CNV detection. ETAAD patients (n = 108, 100% European American, 28% female, average age 20 years, 55% with bicuspid aortic valves) were compared to 7013 dbGAP controls without a history of vascular disease using downsampled Omni 2.5 data. For comparison, 805 sporadic TAAD patients with late onset aortic disease (STAAD cohort) and 192 affected probands from families with at least two affected relatives (FTAAD cohort) from our institution were screened for additional CNVs at these loci with SNP arrays. We identified 47 recurrent CNV regions in the ETAAD, FTAAD and STAAD groups that were absent or extremely rare in controls. Nine rare CNVs that were either very large (>1 Mb) or shared by ETAAD and STAAD or FTAAD patients were also identified. Four rare CNVs involved genes that cause arterial aneurysms when mutated. The largest and most prevalent of the recurrent CNVs were at Xq28 (two duplications and two deletions) and 17q25.1 (three duplications). The percentage of individuals harboring rare CNVs was significantly greater in the ETAAD cohort (32%) than in the FTAAD (23%) or STAAD (17%) cohorts. We identified multiple loci affected by rare CNVs in one-third of ETAAD patients, confirming the genetic heterogeneity of TAAD. Alterations of candidate genes at these loci may contribute to the pathogenesis of TAAD.

## Introduction

Thoracic aortic aneurysms and dissections (TAAD) cause more than 8000 deaths in the United States every year [1]. The annual risk of sudden death from an enlarged thoracic aneurysm due to an acute aortic dissection is more than 10% [2]. Currently, even with the best medical and surgical treatments, 40% of TAAD patients do not survive acute dissections. Timely surgical repair of aneurysms can prevent death. However, there are no clinically available methods to identify patients who are at risk for dissection. While hypertension and bicuspid aortic valves are recognized as common risk factors for TAAD, the genetic alterations predisposing individuals to TAAD remain unknown.

Up to 20% of TAAD patients have an affected relative, and the phenotype is primarily inherited in an autosomal dominant manner characterized by variable expression and incomplete penetrance [3]. Positional cloning approaches using these families led to the identification of inherited mutations in vascular smooth muscle cell (SMC) isoforms of actin (*ACTA2*) and myosin (*MYH11*), which were predicted to disrupt the SMC contraction. Subsequent discoveries of additional genetic variants led us to hypothesize that decreased SMC contractile function may be the underlying cause of the disease [4]. We also demonstrated that the burden of rare copy number variants (CNVs) affecting proteins that interact in a network affecting SMC contraction is greatly increased among patients with no family history, termed sporadic TAAD [5]. In fact, we showed that 16p13.1 duplications, involving *MYH11*, increase the risk for TAAD over ten-fold. These findings are consistent with a genetic model whereby individually rare mutations with relatively large effects on aortic wall structural integrity contribute to disease predisposition [6].

TAAD is associated with a spectrum of congenital LV outflow tract malformations ranging from aortic coarctation and patent ductus arteriosus to subaortic stenosis [7]. The most commonly associated malformation is bicuspid aortic valve (BAV), which is present in 1–2% of the general population. TAAD and BAV can occur together in individuals with chromosomal disorders, such Turner Syndrome, and in children with non-syndromic outflow tract defects, such as tetralogy of Fallot and hypoplastic left heart syndrome. In 10–30% of patients with affected relatives, BAV and TAAD appear to segregate in pedigrees as different manifestations of a single underlying disorder [8]. BAV also occurs six-fold more frequently in the first degree relatives of individuals with sporadic coarctation of the aorta and related cardiovascular malformations [9]. BAV patients are at a greatly increased risk for TAAD with a 70–80% lifetime prevalence of ascending aortic aneurysms and a 6% lifetime incidence of aortic dissections [10]. BAV patients who were enrolled at our institutions with aneurysms or dissections were also significantly younger than patients with tricuspid aortic valves and TAAD. Based on these observations, we propose that BAV and TAAD share common developmental origins and are caused by defects in the same genes or in interacting genes. We therefore investigated the contribution of rare copy number variants to TAAD among younger patients with BAV, coarctation and other LV outflow tract defects. While our conclusions merit some caution due to systematic differences between comparison groups, we demonstrate that this can be an effective approach to identify new candidate genes for congenital heart disease.

## Results

The demographic characteristics of the ETAAD group are shown in Table 1. All patients presented with thoracic aortic aneurysms, primarily involving the aortic root. The mean diameters of the aortic root (mean Z-score 3.2) and ascending aorta (mean Z-score 3.3) exceeded population norms for age and body size by more than three standard deviations. Thirteen subjects developed acute aortic dissections prior to enrollment. Bicuspid aortic valves were significantly more prevalent among ETAAD subjects (55%) than in older STAAD patients (17%). BAV subjects were significantly younger than subjects with tricuspid aortic valves (TAV, 18.4 vs 21.0 years, p = 0.037). The prevalence of Type 1, or right-left, BAV morphology (68%) was similar to population-based cohorts and was not associated with gender or other clinical variables [8]. Aortic Z-scores for the sinuses of Valsalva (n = 88) were not significantly different between BAV and TAV subjects, or between BAV types. However, Z-scores for the ascending aorta (n = 73) were significantly larger in the BAV subgroup (mean 3.7) than in TAV subjects (mean 2.3, P = 0.009). This difference primarily reflected larger ascending diameters in BAV cases with type II or unicuspid morphology (n = 10, mean Z = 4.6) than in BAV cases with type I morphology (n = 20, mean Z = 3.0, P = 0.047).

**Table 1:** Characteristics of 108 Early Onset TAAD Subjects.

	Avg or Num	% or IQR
Age	20	15–25
Female sex	30	(28%)
BAV	59	(55%)
Right-Left or Type I	21	(67%)
Right-Non or Type II	7	(23%)
Unicuspid	3	(10%)
Not evaluable	10	(24%)
No morphology data	18	(31%)
Coarctation	13	(12%)
Dissection	13	(12%)
Surgical repair of aorta	63	(59%)
Z-score: aortic root^a^	3.2	2.3–4.1
Z-score: ascending aorta^b^	3.3	1.4–4.9

BAV: bicuspid aortic valve;

^a^: derived from 88 subjects;

^b^: derived from 73 subjects.

We performed genome-wide CNV analysis of these 108 ETAAD subjects as described above to detect autosomal and X chromosome CNVs (Table 2). After sample-level quality control, 105 individuals were available for analysis. Mean logR ratio standard deviations and wave factors were significantly less in ETAAD cases than in controls (Fig A in S1 File). The number of CNVs called was positively correlated with the logR ratio standard deviation (Fig B in S1 File). CNVs with high confidence scores (PennCNV log Bayes factor > 75 or CNVP confidence score > 250) and CNVs that were identified by at least two algorithms were combined into a consensus call list for further analysis. Q-PCR assays successfully validated 23 of 24 predicted CNV regions (CNVRs, 96%) and confirmed that this approach yields highly reproducible CNVs with few false positive results (Table D in S1 File).

**Table 2:** CNV Discovery in ETAAD Cases and Controls.

	ETAAD (108)		Controls (7013)	
	Autosomes	X chr	Autosomes	X chr
PennCNV	5473 (51)	184 (1.7)	74802 (10.6)	5866 (0.8)
CNVP	699 (6.5)	17 (0.2)	19698 (2.8)	1387 (0.2)
Nexus	4239 (39)	216 (2.0)	42854 (6.1)	12279 (1.8)
Overlap	570 (5.3)	23 (0.2)	12718 (1.8)	1766 (0.3)
Genic CNVs	493 (4.6)	21 (0.2)	9523 (1.4)	1756 (0.3)
Large duplications	114 (1.1)	5	3023 (0.4)	145
Large deletions	44 (0.4)	4	1028 (0.1)	48
Enriched CNVRs in cases	42	4	-	-
Enriched genes	86	19	-	-
In other sporadic or familial cases	10	14		

PennCNV: number of autosomal CNVs detected by PennCNV; CNVP: number of CNVs detected by CNV Partition; Nexus: number of CNVs detected by Nexus Copy Number 7.5. Overlap: number of CNVs detected by all three algorithms; the average number of CNVs per person is shown in parentheses; Large duplications: more than 500 Kb in length; Large deletions: more than 200 Kb in length; CNVRs: CNV regions, including multiple genes.

Within the ETAAD cohort, the total number of CNVs per individual was significantly greater in cases with BAV (n = 59) than in cases with tricuspid aortic valves (n = 49, empiric *P* < 0.05). We then compared CNV rates among ETAAD subjects to STAAD subjects and 6019 control dbGAP genotypes without any history of cardiovascular disorders (Table 3, Table A in S1 File). The overall prevalence of CNVs (P<1x10^-6^), average number of genes spanned by CNVs (P = 0.05) and proportion of CNVs that involve genes (P = 0.007) were all significantly increased in ETAAD subjects. Large (>200 Kb) and rare (<3 events) genic CNVs were significantly more prevalent in ETAAD cases than in the controls and were enriched for genes in the Notch signaling pathway (KEGG hsa04330, empiric *P*<1x10^-5^). The proportion of ETAAD cases with rare CNVs (32%) was significantly greater than that of STAAD cases (17%, chi-squared P = 0.0012). Two large chromosomal anomalies were present in cases, but were also seen in controls: one partial Y chromosome duplication in a phenotypic male, and one mosaic 9p duplication. We concluded that the most deleterious CNV subtypes are significantly enriched in individuals with early onset thoracic aortic disease.

**Table 3:** Distribution of Autosomal CNVs in ETAAD Cases and Controls.

	All			>200 Kb			<3 events			<3 events genic		
	ETAAD	STAAD	Controls	ETAAD	STAAD	Controls	ETAAD	STAAD	Controls	ETAAD	STAAD	Controls
Total autosomal CNVs	503^a^	2395	12825	159^a^	539	4051	203^d^	446	3720	134^f^	278	2411
Deletions	220^a^	826^b^	5299	44^c^	89	1028	105^a^	165	1662	53^a^	75	894
Duplications	283^a^	1569	7526	115^a^	450	3023	98^e^	281	2058	81^g^	203	1517

Number of CNVs in ETAAD (110 early onset TAAD genotypes), STAAD (805 sporadic TAAD genotypes) and controls (6019 genotypes from three dbGAP datasets). ETAAD CNVs, but not STAAD CNVs, were uniformly enriched across all CNV categories;

^a^: P<1x10-5;

^b^: P = 4x10-5;

^c^: P = 5x10-5;

^d^: P = 2x10-5;

^e^: P = 0.001;

^f^: P = 0.004;

^g^: P = 0.003.

P values refer to comparisons between ETAAD or STAAD and controls. CNV rates (CNVs per subject) are presented in Table A in S1 File.

We then performed SNP and region-based tests to identify specific CNVs and genes that are enriched in ETAAD cases. A total of 47 CNVRs containing 105 protein-coding genes were enriched in ETAAD cases (P<0.05) and were rare or absent in dbGAP controls (Table 4). Fifty-nine (56%) of these genes were disrupted by rare CNVs in at least one other cohort with BAV and TAAD. Genetic alterations of *ARSB*, *BGN*, *CXADR*, *EPHA3*, *HIC2*, *HOXA3* and *SUMF1* cause aortic valve defects, aortic developmental abnormalities or TAAD in humans or animal models [11–16]. The most significantly enriched molecular functions of recurrent CNV genes were “embryonic skeletal system development” (GO term 0045995, P = 0.017), most likely due to recurrent deletions of the *HOXA* gene cluster in 7p15.2, and “cysteine-type endopeptidase inhibitor activity” (GO term 0004869, P = 5x10^-5^), reflecting recurrent deletions of cystatin peptidase inhibitor genes in 20p11.21 (*CST1L*, *CST8*, *CST9*, *CST11L*). Cystatins are potent inhibitors of matrix metallproteinases and cathepsins, which degrade elastin in the aortic wall. In a network with known TAAD genes as the seeds, *MAPK1* and *NAP1L1* were ranked as the most significantly interconnected candidate genes. Both genes are expressed in vascular SMCs and interact with central components of the TAAD gene network. Large 22q11 duplications, distal to the DiGeorge syndrome critical region, were observed in one ETAAD case and in two other cases with BAV or LVOTO, but were not present in controls. This CNVR includes several dosage-sensitive candidate genes that are implicated in cardiovascular phenotypes: *BCR*, *HIC2* and *MAPK1*. A region surrounding *BGN* in Xq28 that includes 25 genes was the most highly enriched CNVR in TAAD cases. Duplications of this region were found in two ETAAD and two STAAD cases, but were not present in controls (P = 0.0003, OR 26 (2.9–235)). While the results do not remain significant after adjustment for multiple tests, CNVs involving BGN may contribute to TAAD, because deletion of *BGN* in mice causes thoracic aortic dissections [17]. The other enriched CNVRs do not contain known BAV or TAAD genes.

**Table 4:** Notable Enriched CNVRs in ETAAD Cases.

CNV Region	Type	Genes	Functions
chr1:49861717–49990335	Del	AGBL4	De-glutaminates MYLK
chr2:240290494–240618610	Dup	BC132948, **HDAC4**	Regulator of vascular smooth muscle differentiation
chr3:3671084–3786517	Del	SUMF1	Mutations cause aortic valve defects
chr3:89384566–89417171	Del	EPHA3	Mutations cause aortic valve defects
chr4:139953522–140199813	Dup	C4orf49,CCRN4L,**ELF2**	Potential TAAD biomarker
chr5:78179604–78179604	Dup	ARSB	Mutations cause aortic valve defects
chr7:107196723–107671407	Dup	BCAP29, CBLL1, COG5, DLD, DUS4L, **LAMB1**, **LAMB4**, LOC286002, MIR548, SLC26A3, SLC26A4, SnoU109, U3	Required for angiogenesis and endothelial cell adhesion
chr7:27145024–27317215	Del	AF071167, AK311383, BC035889, DQ655986, EVX1, HOTTIP, HOXA10, HOXA10-HOXA9, HOXA11, HOXA11-AS1, HOXA13, **HOXA3**, **HOXA4**, HOXA5, HOXA6, HOXA7, HOXA9, LOC100133311, MIR196B	Mutations disrupt aortic development
chr8:122327655–122341946	Dup	**HAS2**	Mutations cause cardiac and vascular defects
chr9:46587–503735	Dup	AY343892, AY343902, C9orf66, CBWD1, **DOCK8**, FOXD4, KANK1	Mutations are associated with TAAD
chr12:76417892–76452091	Dup	**NAP1L1**,PHLDA1	Interacts with NOTCH1 to promote cardiac development
chr13:20488212–20524705	Del	ZMYM2	Interacts with TAAD genes SMAD3 and SMAD4
chr15:48701029–48896830	Del	FBN1	Mutated in MFS
chr16:19950501–19979334	Dup	GPRC5B	Mediates retinoic acid signaling in vascular development
chr16:83071614–83099707	Del	CDH13	Implicated in blood pressure regulation and angiogenesis
chr20:23424638–23568490	Del	CST11,CST8,CST9L,CSTL1,CSTT	Deficiency of related genes is implicated in aneurysm progression
chr21:44838661–44854995	Dup	SIK1	Implicated in blood pressure regulation
chr21:18707004–19070558	Dup	BTG3, C21orf37, **CXADR**, TRNA_Gly, Y_RNA	Mutation causes TAAD and cardiac defects
chr22:21458625–24643609	Dup	BCR,**HIC2**, MAPK1, 89 other genes	Mutations case left ventricular outflow tract defects
chrX:152468386–152997097	Dup	MAGEA1, ZNF275, BC018767, ZFP92, TREX2, HAUS7, **BGN**, ATP2B3, FAM58A, DUSP9, SLC6A8, PNCK, BCAP31	Mutation causes TAAD

Selected from a total of 47 enriched CNVRs; chr: chromosome; Dup: disrupting duplication; Del: deletion. All CNVRs were single events with unadjusted *P* = 0.044 for the comparison with controls. The adjusted, study-wide *P* values for all comparisons were 1.0.

## Discussion

Increased copy number burden is associated with greater severity and earlier onset of other complex disorders, including Alzheimer disease, schizophrenia, autism and obesity [18–21]. We previously found that the burden of rare CNVs was significantly greater in FTAAD subjects, whose average age at presentation was 52 years, than in STAAD subjects, whose mean age was 63 years [5]. We acknowledge that there may be differences in the sensitivity and specificity of CNV calls between groups of samples processed by different labs, at different times and using different genomic platforms. Nevertheless, we found that the frequency of rare CNVs in our ETAAD cohort, whose average age was 20 years, was even higher than that of STAAD or FTAAD cases, although the variance of the intensity data, which is directly proportional to the number of CNVs called, was significantly lower in ETAAD cases. Thus, TAAD is another genetic disorder in which the CNV burden is increased in patients with earlier onset disease.

The prevalence of BAV and coarctation in ETAAD subjects provides further evidence that they are genetically predisposed to aortic disease. BAV occurs with coarctation and TAAD in conditions that cause left ventricular outflow tract obstruction, including Shone’s complex and hypoplastic left heart syndrome [22]. These structures are all derived from the fourth pharyngeal arch and appear to be vulnerable to disruption of similar cellular and molecular pathways. However, the relationship between BAV and TAAD is complex and varies significantly depending on the clinical context. In a community-based cohort of older BAV patients, only 25% developed ascending aneurysms over 20 years, with very few aortic dissections [23]. We selected a younger population with TAAD and found that 55% had BAV, a remarkable enrichment that is greater than in cohorts with Turner syndrome (30%) or STAAD (4–17%) [24,25]. We also found that the diameter of the ascending aorta, when corrected for body size, was significantly larger in ETAAD subjects with BAV, even at younger ages. While our results must be interpreted cautiously due to systematic differences between comparison groups, these observations suggest that isolated BAV may constitute one ‘hit’ that, in combination with other genetic factors such as rare CNVs, mutations or chromosomal lesions, predisposes these patients to aggressive aortic disease.

Relevant to this hypothesis, we found that deleterious CNVs are highly enriched in ETAAD, particularly increased in subjects with BAV, and contain genes that are known to cause BAV and TAAD when mutated. Specific CNVRs that are recurrent in early onset and sporadic TAAD cases are likely to contain new candidate genes that can be prioritized by their expression in aortic tissues or interaction with pathways that contribute to aortic development or structural integrity. Pathways affected by genes in ETAAD-enriched CNVs include genes mutated in disorders that cause aortic valve defects or arterial aneurysms (*DOCK8*, *ARSB*), genes involved in LVOT and aortic development (*LIMS3*, *DTX2*, *PTCH1*, *HOXA3*), genes that maintain the integrity of vascular smooth muscle cell interactions with the extracellular matrix (*LAMB1*, *BGN*, *FBN1*, *CDH13*) and genes that regulate ECM proteolysis (*CTSL2*, *cystatins*). Intriguingly, *RPL38*, one of 17 genes involved in large recurrent 17q25.1 duplications, was shown to regulate the translation of the same *HOXA* genes that are affected by recurrent deletions in TAAD subjects. We also found that deletions and duplications of the same regions are associated with TAAD, indicating that indicating that aortic development may be exquisitely sensitive to perturbations of the expression of genes in these regions. These results are similar to our genome-wide CNV analysis of STAAD and FTAAD cases, and highlight the multiple disease mechanisms that may lead to TAAD.

Strengths of this study included comprehensive comparisons of rare CNVs across diverse groups with a greatly increased prevalence of BAV and TAAD, which led us to identify overlapping genomic signals for BAV. Limitations included the use of multiple genotyping platforms for CNV detection, which limited our ability to compare CNV rates between groups, potential selection bias related to the recruitment of study subjects at specialized centers, and inadequate statistical power to identify associations between CNV prevalence or burden with BAV or TAAD separately or with outcomes. We also do not have data from trios to confirm that potentially pathogenic CNVs are *de novo* events in affected individuals and are therefore uncertain about their penetrance or true association with disease.

Our findings confirm the hypothesis that the prevalence of large deleterious CNVs involving cardiac or vascular developmental genes is significantly increased in ETAAD. BAV and TAAD may therefore be grouped with other developmental disorders such as autism and schizophrenia whose penetrance and severity are determined in part by rare CNVs. ETAAD patients are at high risk for complications and/or death from TAAD. We propose that microarray-based tests to detect rare CNVs should be considered in the clinical evaluation of ETAAD patients to assist decisions about counseling, familial screening or interventions.

## Materials and Methods

The Committee for the Protection of Human Subjects (CPHS) at the University of Texas Health Science Center at Houston granted permission for this work (protocols HSC-MS-07-0399 and HSC-MS-01-251) and approved all informed consent procedures. Participants provided their written informed consent to participate in this study. Included cases were non-Hispanic patients of European descent with ascending aortic aneurysms or thoracic aortic dissections (TAAD) from our institution and the National Registry of Genetically Triggered Thoracic Aortic Aneurysms and Other Cardiovascular Conditions (GenTAC). In all subjects, the diagnosis was confirmed by echocardiography, computed tomography or magnetic resonance imaging. Aortic measurements were obtained and BAV morphologies were classified according to published criteria [23]. We excluded ETAAD subjects who had any primary relatives with BAV or TAAD or were more than 30 years of age at the time of diagnosis. Patients with aortic lesions associated with trauma, infection, aortitis, syndromic forms of TAAD, sex chromosome aneuploidy or connective tissue disorders; patients with an isolated intramural hematoma, penetrating aortic ulcer, or pseudo-aneurysm; and patients who received packed red blood cell, whole blood or platelet transfusions within 72 hours of blood collection were excluded.

To identify recurrent CNVs, ETAAD genotypes were systematically compared with CNV data from three other cohorts with BAV and TAAD: 805 sporadic TAAD cases (STAAD), as well as affected probands from families with inherited TAAD (FTAAD) or inherited left ventricular outflow tract obstructive defects (LVOTO). The characteristics of the STAAD and LVOTO cohorts were previously described [5,26].

The primary controls for the study were Illumina genotypes of 7013 subjects in three cohorts obtained from the Database of Genotypes and Phenotypes (dbGAP, Table B in S1 File). Our analysis was confined to unrelated individuals of European descent from each dataset. All control genotypes were derived from analysis of whole blood. After appropriate quality controls, 6019 individuals were available for comparison. Phenotypic data relevant to TAAD were not available from any of the control samples used.

DNA was extracted from whole blood samples collected from each patient at enrollment and stored at -80°C. The quantity of double-stranded DNA was measured using PicoGreen (Invitrogen Corporation, Carlsbad, CA). Samples with a DNA concentration below 40μg/ml were concentrated using a SpeedVac system; samples with a DNA concentration above 150μg/ml were normalized to 100μg/ml. Purity of the DNA samples was assessed using a NanoDrop spectrophotometer (Thermo Scientific, Inc., Wilmington, DE).

**Fig 1:**
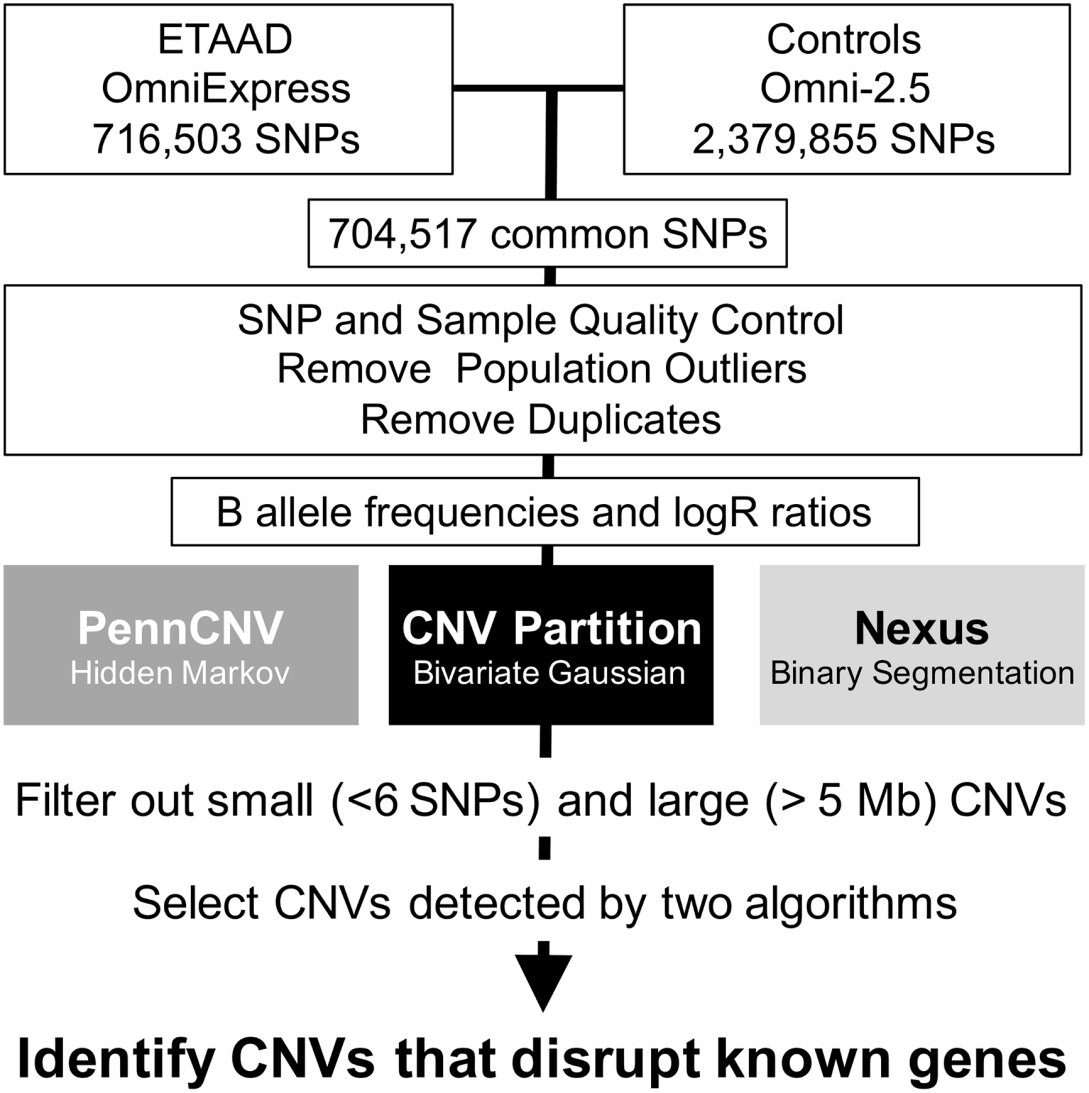
CNV Analysis Workflow.

Samples were genotyped on HumanOmniExpress beadchips (Illumina, Inc., San Diego, CA) with appropriate quality standards for labeling, single base extension, hybridization, stringency and non-specific binding according to our previously published methods. Allele detection and genotype calling were performed in the GenomeStudio genotyping module (v 2011.1, Illumina, Inc.) using updated manifests in the hg19 genome build with 716,503 SNPs (manifest humanomniexpress-24v1-0_a, 15 samples) or 730,525 SNPs (manifest humanomniexpress-12v1_j, 93 samples). All SNPs in humanomniexpress-24 beadchips are also included in humanomniexpress-12 beadchips, which have an additional 14,022 SNPs that are not present in humanomniexpress-24. To equalize SNP densities between cases and controls, both the control Omni-2.5 panels (2,379,855 SNPs) and the Omni-Express panels were filtered to select only the subset of SNPs that overlap with OmniExpress arrays (704,517) prior to the analysis (Fig 1). Samples that did not cluster with HapMap CEU (Utah residents with ancestry from northern and western Europe) samples in multidimensional scaling analysis, replicate samples, unexpected duplicates or samples with mismatched gender, excess heterozygosity, excess homozygosity or more than 2% missing genotypes were excluded from further analysis. SNPs with call frequency < 0.98, cluster separation < 0.25 or heterozygote excess < -0.5 or >0.5 were also excluded.

B allele frequencies (BAF) and logR ratios were exported from GenomeStudio in a single tab-delimited report file for analysis by PennCNV (v. 2011) [27] and Nexus Copy Number (v. 7.5, Biodiscovery, Inc., Hawthorne, CA). CNVPartition was run as a plug-in within GenomeStudio. For all three algorithms, CNVs with fewer than six contiguous probes and CNVs less than 20 kilobases or > 5 megabases in length were excluded. For PennCNV analysis, the report file was split into individual sample files before CNVs were called using a custom-generated population frequency of B allele (.pfb) file. For Nexus analysis, the SNPRank algorithm was used with significance threshold of 1x10^-8^, default gender set to female and systematic correction based on array type. Sample-level quality control analysis was performed using PennCNV. Samples were excluded from further analysis if any of the following criteria were met: standard deviation of logR ratios > 0.35 for autosomes or 0.45 for the X chromosome, B allele frequency drift > 0.1, waviness factor > 0.05 or number of CNVs identified > 2 standard deviations above the mean of each dataset. Small adjacent CNVs were merged into larger contiguous CNVs using the ‘clean_cnv’ perl script in the PennCNV package. CNVs in pericentromeric, subtelomeric, pseudo-autosomal and immunoglobulin regions and regions with allelic imbalance or loss of heterozygosity were also excluded. The frequencies that genotypes were excluded were not significantly different between datasets.

We used the ‘cnv-check-no-overlap’ function in PLINK (v 1.07) with default overlap set at 50% to identify CNVs that were called by at least two algorithms and merged the output of all pairwise comparisons into a single file [28]. We then combined these overlapping CNV calls with the highest-confidence CNVs from CNVPartition (confidence score > 250) and PennCNV (log Bayes factor > 75) to generate the final CNV lists for analysis. CNVs with fewer than three total occurrences in cases and controls were classified as rare CNVs. CNVRs were defined as the intersection of overlapping CNV calls, and CNVRs with at least 50% overlap were considered to be recurrent. CNV annotation functions in PLINK (—cnv-freq and —cnv-report-regions) were used to determine CNV frequencies and to identify genes within TAAD-associated CNVs. Gene lists were then compared to identify recurrent CNVs within and across groups using standard functions in Unix. Gene enrichment in rare CNVs was determined using methods derived from Raychaudhuri et al., and burden tests were permuted to generate empiric P-values [29]. The burdens of deletions (copy number 0 and 1) and duplications (copy number 3 and 4) were analyzed separately. Case-control comparisons of individual CNVRs were evaluated using Fisher exact tests. For functional analysis, gene lists were entered into the ToppGene Analysis Suite to generate permuted P values for enriched pathways and functions [30]. All candidate CNVs were manually reviewed and verified in GenomeStudio.

A subset of 24 randomly selected CNVRs was independently validated using quantitative real-time PCR (Q-PCR) with SYBR Green as described [31]. Primers (Table C in S1 File) were designed using Primer BLAST (NCBI) and purchased from Eurofin MWG Operon (Huntsville, AL). Reactions were run on a Vii A7 Fast Real-Time PCR System (Applied Biosystems, Foster City, CA) with six replicates and analyzed using Sequence Detection Software (Applied Biosystems). Relative copy number was calculated from Ct values using a custom Excel spreadsheet as described [32].

Functional analysis and candidate gene prioritization were carried out using the ToppGene Suite (toppgene.cchmc.org) and the STRING protein interaction database (string-db.org). The training datasets for ToppGene consisted of STRING interaction networks that were built using known TAAD or BAV genes as the seeds after excluding interactions that were not experimentally validated. The significance of associations between the top 50 ranked CNV genes and biological pathways was measured as a ratio of the number of genes that map to the pathway divided by total number of genes within the pathway and estimated by permutation using 1600 randomly selected genes. Benjamini-Hochberg analysis with a false discovery rate of less than 0.05 was used to adjust for multiple testing. Network interactions were algorithmically generated based on their connectivity to known human TAAD genes. Network interaction scores were derived from k-step Markov modeling using a step size of 6 and neighborhood distance of 1. Categorical values were compared using Wilcoxon signed-rank tests.

## Supporting Information

S1 File Supporting Information. Part A, Supplemental Acknowledgements. Fig A, Distributions of LogR Ratio Standard Deviations and Wave Factors. Fig B, LogR Ratio Standard Deviations vs. Number of CNVs Called. Table A, Rates of Autosomal CNVs in ETAAD Cases and Controls. Table B, Control Datasets Used for Analysis. Table C, Q-PCR Primers Used for CNV Validation. Table D, Q-PCR Validation Results for Rare CNVs.
(DOCX)
