# Supplementary material for: Recurrent Rare Genomic Copy Number Variants and Bicuspid Aortic Valve Are Enriched in Early Onset Thoracic Aortic Aneurysms and Dissections
Source: PLoS One. 2016 Apr 19;11(4):e0153543. doi: 10.1371/journal.pone.0153543 (PMC4836726; doi:10.1371/journal.pone.0153543)
Supplement: S1 File — Part A, Supplemental Acknowledgements. Fig A, Distributions of LogR Ratio Standard Deviations and Wave Factors. Fig B, LogR Ratio Standard Deviations vs. Number of CNVs Called. Table A, Rates of Autosomal CNVs in ETAAD Cases and Controls. Table B, Control Datasets Used for Analysis. Table C, Q-PCR Primers Used for CNV Validation. Table D, Q-PCR Validation Results for Rare CNVs. (DOCX) [file pone.0153543.s001.docx]

**Part A. Supplemental Acknowledgments**

The GenTAC Registry Investigators are:

Johns Hopkins University

Williams Ravekes, M.D.

Harry C. Dietz, M.D., Ph.D.

Kathryn W. Holmes, M.D.

Jennifer Habashi, MD

University of Texas - Houston

Dianna M. Milewicz, M.D. Ph.D.

Siddharth K. Prakash, M.D., Ph.D

Baylor College of Medicine

Scott A. LeMaire. M.D.

Shaine A. Morris, M.D.

Oregon Health & Science University

Cheryl L Maslen, Ph.D.

Howard K. Song, M.D., Ph.D

G. Michael Silberbach, M.D.

University of Pennsylvania

Reed E. Pyeritz, M.D., Ph.D.

Joseph E. Bavaria M.D.

Karianna Milewski, M.D., Ph.D.

Weill Medical College of Cornell University

Richard B. Devereux, M.D., Ph.D.

Jonathan W. Weinsaft, M.D.

Mary J. Roman, M.D.

The Queen’s Medical Center

Ralph Shohet, M.D.

National Institute on Aging

Nazli McDonnell, M.D.

MedStar Health Research Institute

Federico M. Asch, M.D.

University of Michigan

Kim A. Eagle, M.D.

National Heart, Lung, and Blood Institute

H. Eser Tolunay, Ph.D.

Patrice Desvigne-Nickens, M.D.

National Institute of Arthritis, Musculoskeletal and Skin Diseases

Hung Tseng, PhD

RTI International

Barbara L. Kroner, Ph.D.

**Figure A. Distributions of LogR Ratio Standard Deviations** **and Wave Factors**


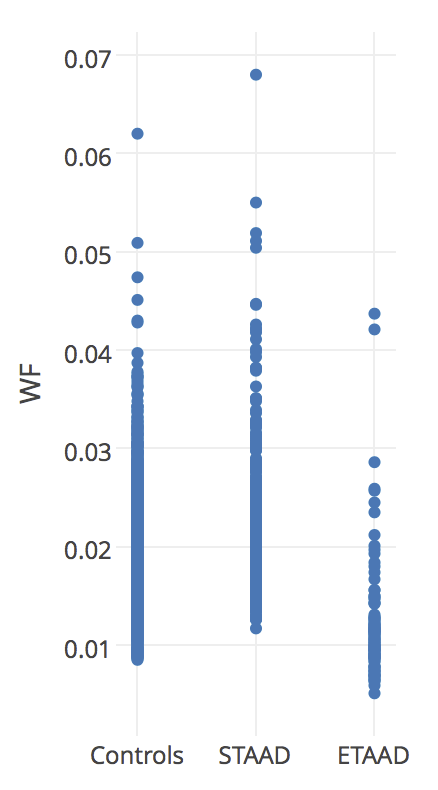

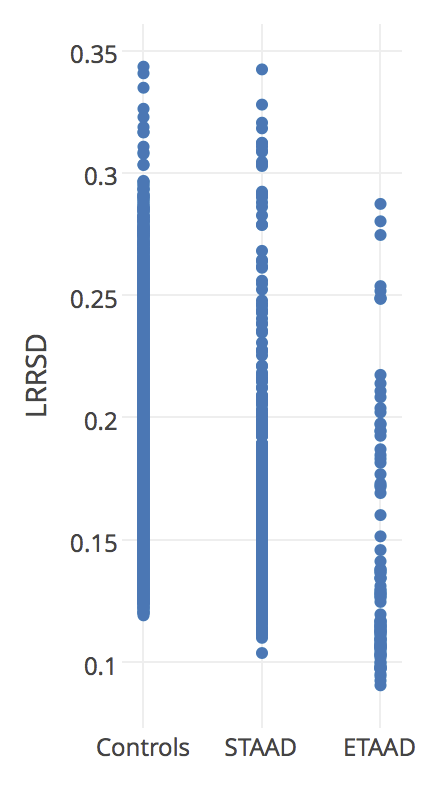


**Supplementary Figure 1.** Distributions of standard deviations of logR ratios (LRRSD) and absolute values of wave factors (WF) of the early onset TAAD (ETAAD) cohort, sporadic TAAD (STAAD) cohort and dbGAP controls. Black bars and lines indicate mean values and interquartile distances. Kruskal-Wallis *P*-values for the overall significance of both comparisons were 0.0001. *P*-values for all Dunn’s pairwise comparisons were less than 0.01, except for the comparison between STAAD and ETAAD LRRSD values (*P*=0.02).

| **Table A. Rates of Autosomal CNVs in ETAAD Cases and Controls** | | | | | | | | | | | | |  |
| --- | --- | --- | --- | --- | --- | --- | --- | --- | --- | --- | --- | --- | --- |
|  | **All** | | | **>200 Kb** | | | **<3 events** | | | **<3 events genic** | | | |
|  | ETAAD | STAAD | Controls | ETAAD | STAAD | Controls | ETAAD | STAAD | Controls | ETAAD | STAAD | | Controls |
| Total autosomal CNVs | 5.08^a^ | 1.94 | 2.11 | 1.34^a^ | 0.67 | 0.67 | 0.74^d^ | 0.28 | 0.38 | 0.44^f^ | 0.20 | | 0.28 |
| Deletions | 1.82^a^ | 1.03^b^ | 0.87 | 0.39^c^ | 0.11 | 0.17 | 0.64^a^ | 0.15 | 0.25 | 0.36^a^ | 0.09 | | 0.14 |
| Duplications | 3.26^a^ | 0.92 | 1.24 | 0.95^a^ | 0.56 | 0.50 | 0.44^e^ | 0.22 | 0.25 | 0.35^g^ | 0.17 | | 0.19 |
| Average number of CNVs per individual in ETAAD (110 early onset TAAD genotypes), STAAD (805 sporadic TAAD genotypes) and controls (6019 genotypes from three dbGAP datasets). ETAAD CNV rates were uniformly increased across all CNV categories vs STAAD or controls. Except for total deletions, STAAD CNV rates were not significantly different from controls; a: *P*<1x10^-5^; b: *P*=4x10^-5^; c: *P*=5x10^-5^; d: *P*=2x10^-5^; e: *P*=0.001; f: *P*=0.004; g: *P*=0.003. *P* values refer to comparisons between ETAAD or STAAD and controls. | | | | | | | | | | | | | |
|  |  |  |  |  |  |  |  |  |  |  |  |  |  |
|  |  |  |  |  |  |  |  |  |  |  |  |  |  |
|  |  |  |  |  |  |  |  |  |  |  |  |  |  |

| **Table B. Control Datasets Used for Analysis** | | | |
| --- | --- | --- | --- |
| **Name** | **Accession** | **Total n** | **Used n** |
| Genetic Epidemiology of Refractive Error in KORA (Kooperative Gesundheitsforschung in der Region Augsburg) | phs000303.v1.p1 | 1869 | 1865 |
| The Genetic Architecture of Smoking and Smoking Cessation | phs000404.v1.p1 | 1525 | 935 |
| A Genome-Wide Association Study of Fuchs' Endothelial Corneal Dystrophy (FECD) | phs000421.v1.p1 | 3619 | 3218 |
|  |  | 7013 | 6018 |

| **Table C. Q-PCR Primers Used for CNV Validation** | | |
| --- | --- | --- |
| **Gene** | **Forward Primer** | **Reverse Primer** |
| ABCC1 | TCAGCAGGAAACAGCAAGACAGG | AGCAGCAACTGATGGGACAAAGG |
| ADAM12 | TTCCTGCTGGCATGTTAGTCTTGG | AGTGGATGTTGGCCTTGCTTCCC |
| AKAP13 | ATGACAGGTGGTGGTCGTGTGG | TCTGATGTGTGGGAGTGGAGGAGG |
| ARSB | AAGCAGCCTTGACACAGACACCC | GGGCTTTGTGGAGGAGCTGAAACC |
| BCAP29 | GGATGTACCCGAACCTGTTGCC | GTTTGTCATGTTAGCAGGGCTGGG |
| BGN | TGCTCACAAGCATGGACTGAACC | CTTGTTGTTCACCAGGACGAGGG |
| BRCC3 | CCGCCTGACACTTGTTTAGTCTCC | ACTCCAGTGTCTTTCTGACCTGCC |
| C3 | GCAGTTGAGCCCTGGATTGTGACC | AGTCCATTGGCTCTGTCCTCTCCC |
| C9OR66 | GAATAATGAAGCAGGCGAGGACGG | GCCACGGAGTGTCTCATAAACGG |
| CHL1 | AGCGATGTTCATGGAGTTTGTGCC | TTCCGAGTCAGCACCCTCTACC |
| CLSTN2 | AGCAGGTCTAGGGACCACAAGG | AGCAAGGTCCGTTTCCCTCACC |
| CNTN6 | GCCTCTGGTCTGCTAGTCTGTTGG | AGCAGAGACCTTGACCTGAACCC |
| CRKL | ATCCACTACCTGGACACCACCACC | TGCGGGGAATATGGGTTCAAGCG |
| CTSB | TTTCTTGTTGGCTCTGGTGCTGG | AATGACCAAAGACGAAGGGAAGCC |
| CXADR | GGGTTGAGCATAGGAGCAAACAGG | GGTGCTCAGGAGGATGGTAACAGG |
| CYTSA | ACCACCTTGTCCACGTCACATGC | AGGTCACAGTGCAACTCCTGGC |
| DOCK8 | GACCTTCTGGTCTGAAACGCTGG | AACTTCTCCAGGTGGTTGTCCTGC |
| ERICH1 | TGTGCTTTGAGGCTGTGCTTGGG | CACTGTTTAGCCTGACTGCCAACC |
| AM58 | TGCGCTTGCTCGTAAACCTCACC | TGAACGATGACTGGGTGAGTCTGG |
| BN1 | TTGTGCTTCTCCGTTTCCTGCC | TGTGTTTCTGGAATGGGCATGGG |
| FH | TATGCAGAGACTGGAGCCTTGGG | AATCTCCAAGACACCCTCCAACCC |
| FOXM1 | GTAGGTATAGGAGGCTGTGCTCCC | AGGCTCCAATTCCAACCCATGCC |
| GJA3 | TTCTTTGCTCCGTGGTCCTGCC | CCTGAGCTGGAAGAAAGGTGAGGG |
| GPRC5C | TACGCTTGGTGACTGGTGGAGG | CGCACACTCAAGACAGAGTCCG |
| HS3ST3B1 | CAAAGCGAGCCACCCTCTAACC | AAGTTCCTTGAGGGCGGAGACC |
| IGBPL1 | TTCCCAAGGTTTGTCACTTGTCCC | CACTTGCACACCCACCAAAGGG |
| ISM1 | GCGTGTGGGATTGTCATCTACTCC | AGTCAGGCCAACAGGAAGAAGGG |
| KIAA1529 | CTCTCTCCCTCCAATTCTCCTCCC | TCACTCTGCCATGCTCTTCGGG |
| KSR1 | GAGTGCCTGGAAATAGCCTGGTGG | CATGCAAGGAACCCAAGGTGCTGG |
| LAMA4 | TGGAAGAATGGCAAGTGAGCGTCC | TGGAGAAGACAAGGAGGGAGCTGG |
| LAMB1 | AAAGGTGCGATTAGTGAGCAGGG | ACAGGAGCAAAGAGGGTGATGGG |
| LRP6 | TGAGAGAAGAGAACGCGAGAAGGG | ACACATACAACAAGGCCACCTCCC |
| MYH11 | GATGAGGTGGTTGTGGAGCTGG | CTCAGGTTGTGTAGCACGGAGG |
| PCSK6 | GTCCGGGCCTTTGACATTCAGG | GCAGGTCAGGAAGCAGCACTTTGG |
| PDLIM7 | AGCTTGCAGTCTTGGCTGTGGG | GCCCGCCTCACTCATTCCATTTCC |
| PMP22 | GGGATAATTGGCAGCAGAAGCACC | AATCGCTATGGCCTACCCAGCC |
| PRKCE1 | TGTAGTCTGCGGGTGTATGTGGG | TGTTGCATGGTCCTTTGGGCAGG |
| PRMT8 | AGTGATGGAGGTGGGCAAGGATGG | CTGGGTACTTCGCTTTGTGGAGGG |
| PTCH1 | GCCGGGTTCATTGTGTTTACGAGG | ACAGCCGAGTGCAAAGGGAAGG |
| PTPN18 | ACACATGGGCTGTGCAATCAGGG | CTAGCCCAGCTTCAGCATACTTCC |
| RAB36 | AGGTCAGATGCGGGAAGACAACC | TGAAAACCCTCACTTCACCAGGC |
| RPL38 | AAGTGTTGGAGCCGTGCTTATCC | GGACAGGCTTGCACAGATAGTTGG |
| SMYD3 | GCAAGTGGACAGCATCTAGGAAGG | TGGGACACATTTGGGTGGAGGG |
| SPTLC3 | TTCAAGCCAACGCCAAGTTCCC | TTCGGTCATACCTAGCTTCCCTCC |
| TJP2 | TCTGACTCTCCTCTGCCCTTTCCC | GAATTTGGCAGCACCACCAGCC |
| TRIM60 | CCGCGAGAGAATGAACGGATGG | GGCAGCATGGTTGGGATTGATGG |
| TRIM61 | TTTGCTGATCCGTCTGCTCCAGG | CACTAAGGAGATCCAGTCGTGGGC |
| TSPAN9 | ATGTCGAAGGGTCCTCCTCACC | AGCACCTACGGAATAGGCATGGG |
| WDR64 | CTGACCATTTGCTCCTTCCTGCCC | TGGCTGTATCTTGGAGGGTGTGG |

| **Table D. Q-PCR Validation Results for Rare CNVs** | | | | | | | | | | | | |
| --- | --- | --- | --- | --- | --- | --- | --- | --- | --- | --- | --- | --- |
| **TRIM61** |  |  |  |  |  |  |  |  |  | | | |
| **CtR Avg** | **CtR SD** | **SEMr** | **_∆CTq_** | **∆CqTavg** | **SEM∆CT** | **∆∆CTq** | **SEM∆∆CTq** | **RCN** | **95% CI** | | | |
| 29.81 | 0.0725 | 0.0362 | _-0.82_ | -0.86175 | 0.17760623 | 0 | 0.25117314 | 2 | 1.68042582 | 2.38034904 | | |
| 30.04 | 0.2082 | 0.1041 | _-1.008_ | -0.981 | 0.14665838 | -0.1192 | 0.23033162 | 2.17234 | 1.8517868 | 2.54838146 | | |
| 29.93 | 0.1298 | 0.0649 | _-1.365_ | -1.417 | 0.06598699 | -0.5553 | 0.18946835 | 2.938847 | 2.57715931 | 3.35129438 | | |
| 30.73 | 0.1683 | 0.0841 | _-0.606_ | -0.573 | 0.39082413 | 0.2888 | 0.42928717 | 1.637222 | 1.21584773 | 2.20463054 | | |
| 30.03 | 0.0843 | 0.0421 | _-2.196_ | -1.48425 | 0.29752459 | -0.6225 | 0.34650376 | 3.07908 | 2.42165912 | 3.91497562 | | |
| 30.65 | 0.391 | 0.1955 | _-0.242_ | -0.7415 | 0.26554168 | 0.1203 | 0.31946261 | 1.840056 | 1.4745625 | 2.21617416 | | |
|  |  |  |  |  |  |  |  |  |  |  | | |
| **TRIM60** |  |  |  |  |  |  |  |  |  | | | |
| **CtR Avg** | **CtR SD** | **SEMr** | **_∆CTq_** | **∆CqTavg** | **SEM∆CT** | **∆∆CTq** | **SEM∆∆CTq** | **RCN** | **95% CI** | | | |
| 27.28 | 0.1547 | 0.0773 | _2.598_ | 2.529 | 0.0853932 | 0.0005 | 0.15103834 | 1.999307 | 1.80057957 | 2.21996672 | | |
| 27.51 | 0.1369 | 0.0684 | _2.383_ | 2.5285 | 0.12458163 | 0 | 0.17618504 | 2 | 1.77008049 | 2.25978425 | | |
| 27.52 | 0.073 | 0.0365 | _2.273_ | 2.46275 | 0.05277508 | -0.0657 | 0.1352989 | 2.093257 | 1.90587073 | 2.2990663 | | |
| 28.73 | 0.1146 | 0.0573 | _1.585_ | 1.79725 | 0.08676652 | -0.7312 | 0.15181901 | 3.320153 | 2.98851916 | 3.68858843 | | |
| 27.56 | 0.23 | 0.115 | _2.5_ | 2.49075 | 0.12376262 | -0.0377 | 0.17560686 | 2.053023 | 1.81773616 | 2.31876487 | | |
| 27.73 | 0.0943 | 0.0471 | _2.683_ | 2.476 | 0.1201001 | -0.0525 | 0.17304513 | 2.07412 | 1.8396795 | 2.24889861 | | |
|  |  |  |  |  |  |  |  |  |  |  | | |
| **RPL38** |  |  |  |  |  |  |  |  |  | | | |
| **CtR Avg** | **CtR SD** | **SEMr** | **_∆CTq_** | **∆CqTavg** | **SEM∆CT** | **∆∆CTq** | **SEM∆∆CTq** | **RCN** | **95% CI** | | | |
| 30.37 | 0.1588 | 0.0794 | _-2.648_ | -2.54 | 0.0910698 | -0.0115 | 0.1543188 | 2.016006 | 1.8114958 | 2.24360556 | | |
| 29.81 | 0.0725 | 0.0362 | _-2.398_ | -2.479 | 0.08381725 | 0.0495 | 0.15015297 | 1.932543 | 1.74152034 | 2.14451777 | | |
| 30.04 | 0.2082 | 0.1041 | _-2.383_ | -2.5285 | 0.12458163 | 0 | 0.17618504 | 2 | 1.77008049 | 2.25978425 | | |
| 29.98 | 0.0763 | 0.0381 | _-2.473_ | -2.53775 | 0.059247 | -0.0093 | 0.13795213 | 2.012866 | 1.82930889 | 2.21484074 | | |
| 30.53 | 0.1303 | 0.0652 | _-1.585_ | -1.79725 | 0.08676652 | 0.7312 | 0.15181901 | 1.204764 | 1.08442567 | 1.33845553 | | |
| 30.03 | 0.0843 | 0.0421 | _-2.7_ | -2.51575 | 0.12065995 | 0.0127 | 0.17343416 | 1.982403 | 1.75785512 | 2.23563472 | | |
| 30.18 | 0.2056 | 0.1028 | _-2.383_ | -2.401 | 0.11361045 | 0.1275 | 0.1686058 | 1.830833 | 1.62889614 | 2.04047136 | | |
|  |  |  |  |  |  |  |  |  |  |  | | |
| **FOXM1** |  |  |  |  |  |  |  |  |  | | | |
| **CtR Avg** | **CtR SD** | **SEMr** | **_∆CTq_** | **∆CqTavg** | SEM∆CT | **∆∆CTq** | **SEM∆∆CTq** | **RCN** | **95% CI** | | | |
| 28.88 | 0.2926 | 0.1463 | _-0.37_ | -0.06725 | 0.25828241 | 0 | 0.36526649 | 2 | 1.55265092 | 2.57623909 | | |
| 29.19 | 0.1294 | 0.0647 | _-0.41_ | -0.22625 | 0.13336258 | -0.159 | 0.2906809 | 2.233027 | 1.82553534 | 2.73147721 | | |
| 28.45 | 0.0623 | 0.0312 | _-0.555_ | -0.0815 | 0.36505658 | -0.0143 | 0.44718688 | 2.019854 | 1.48150529 | 2.75382626 | | |
| 28.73 | 0.3839 | 0.1919 | _-1.076_ | -0.73125 | 0.19381052 | -0.664 | 0.32291225 | 3.16894 | 2.53342298 | 3.96387778 | | |
| 30.2 | 0.2902 | 0.1451 | _-0.626_ | 0.03325 | 0.195256 | 0.1005 | 0.32378189 | 1.865419 | 1.49041914 | 2.33477233 | | |
| 28.47 | 0.4722 | 0.2361 | _-1.187_ | -0.225 | 0.30345656 | -0.1578 | 0.39849177 | 2.231093 | 1.69262074 | 2.9408685 | | |
| 28.66 | 0.185 | 0.0925 | _-0.256_ | -0.05775 | 0.13291158 | 0.0095 | 0.29047425 | 1.986875 | 1.62453472 | 2.32143327 | | |
|  |  |  |  |  |  |  |  |  |  |  | | |
| **DOCK8** |  |  |  |  |  |  |  |  |  | | | |
| **CtR Avg** | **CtR SD** | **SEMr** | **_∆CTq_** | **∆CqTavg** | **SEM∆CT** | **∆∆CTq** | **SEM∆∆CTq** | **RCN** | **95% CI** | | | |
| 28.88 | 0.2926 | 0.1463 | _-0.13_ | 0.20175 | 0.15598431 | 0 | 0.22059513 | 2 | 1.71642268 | 2.3304283 | | |
| 29.22 | 0.0817 | 0.0408 | _-0.228_ | 0.080249 | 0.1426999 | -0.1215 | 0.21141043 | 2.175732 | 1.87916368 | 2.51910557 | | |
| 28.4 | 0.1078 | 0.0539 | _0.173_ | 0.1065 | 0.15870489 | -0.0953 | 0.22252718 | 2.136502 | 1.83111642 | 2.49281857 | | |
| 29.03 | 0.2254 | 0.1127 | _-0.262_ | 0.157751 | 0.12625281 | -0.044 | 0.20067605 | 2.061936 | 1.79417874 | 2.36965285 | | |
| 30.2 | 0.2902 | 0.1451 | _-0.234_ | 0.380501 | 0.3211588 | 0.1788 | 0.35703513 | 1.766936 | 1.37956607 | 2.26307564 | | |
| 28.67 | 0.8478 | 0.4239 | _-1.566_ | -0.47725 | 0.44828137 | -0.679 | 0.47464439 | 3.20206 | 2.30434382 | 4.44950475 | | |
| 28.51 | 0.1765 | 0.0882 | _-0.853_ | -0.55575 | 0.09424419 | -0.7575 | 0.18224454 | 3.381119 | 2.97988407 | 3.77948467 | | |
|  |  |  |  |  |  |  |  |  |  |  | | |
| **CXADR** |  |  |  |  |  |  |  |  |  | | | |
| **CtR Avg** | **CtR SD** | **SEMr** | **_∆CTq_** | **∆CqTavg** | **SEM∆CT** | **∆∆CTq** | **SEM∆∆CTq** | **RCN** | **95% CI** | | | |
| 29.08 | 0.1081 | 0.0541 | _0.13_ | -0.20175 | 0.15598431 | 0 | 0.22059513 | 2 | 1.71642268 | 2.3304283 | | |
| 29.65 | 0.2302 | 0.1151 | _-0.272_ | -0.13025 | 0.16213668 | 0.0715 | 0.22498758 | 1.903295 | 1.62846336 | 2.2245082 | | |
| 28.88 | 0.0616 | 0.0308 | _0.027_ | -0.2065 | 0.12177383 | -0.0047 | 0.19788879 | 2.006595 | 1.74940038 | 2.30160153 | | |
| 29.19 | 0.1138 | 0.0569 | _0.262_ | -0.15775 | 0.12625281 | 0.044 | 0.20067605 | 1.939924 | 1.68801097 | 2.22943228 | | |
| 31.03 | 0.2739 | 0.137 | _-0.466_ | -0.8305 | 0.19952345 | -0.6288 | 0.25326016 | 3.09245 | 2.59456047 | 3.68588354 | | |
| 28.54 | 0.1619 | 0.081 | _0.566_ | -0.12275 | 0.2093194 | 0.079 | 0.26104735 | 1.893427 | 1.58003036 | 2.26898484 | | |
| 28.56 | 0.3718 | 0.1859 | _-0.147_ | -0.04425 | 0.20578794 | 0.1575 | 0.25822428 | 1.793154 | 1.49928533 | 2.10279923 | | |
|  |  |  |  |  |  |  |  |  |  |  | | |
| **BRCC3** |  |  |  |  |  |  |  |  |  | | | |
| **CtR Avg** | **CtR SD** | **SEMr** | **_∆CTq_** | **∆CqTavg** | **SEM∆CT** | **∆∆CTq** | **SEM∆∆CTq** | **RCN** | **95% CI** | | | |
| 28.66 | 0.1228 | 0.0614 | _3.544_ | 3.6295 | 0.09013839 | 0.0472 | 0.26097266 | 1.935559 | 1.61527226 | 2.31935342 | | |
| 28.42 | 0.1299 | 0.065 | _2.966_ | 3.5825 | 0.42591078 | 0.0002 | 0.49130621 | 1.999654 | 1.42251525 | 2.81094755 | | |
| 28.18 | 0.1533 | 0.0766 | _2.963_ | 3.6175 | 0.26615621 | 0.0353 | 0.36169175 | 1.951725 | 1.51893261 | 2.50783317 | | |
| 28.52 | 0.1288 | 0.0644 | _3.445_ | 3.760001 | 0.13809081 | 0.1778 | 0.28115986 | 1.768161 | 1.4550711 | 2.14861853 | | |
| 29.08 | 0.1572 | 0.0786 | _3.172_ | 2.97825 | 0.2280891 | -0.604 | 0.33467362 | 3.039851 | 2.41049068 | 3.83353167 | | |
| 28.6 | 0.0889 | 0.0445 | _3.53_ | 3.58225 | **0.24491182** | 0 | 0.34635761 | 2 | 1.5731349 | 2.413868 | | |
|  |  |  |  |  |  |  |  |  |  |  | | |
| **BCAP29** |  |  |  |  |  |  |  |  |  | | | |
| **CtR Avg** | **CtR SD** | **SEMr** | **_∆CTq_** | **∆CqTavg** | **SEM∆CT** | **∆∆CTq** | **SEM∆∆CTq** | **RCN** | **95% CI** | | | |
| 32.49 | 0.3588 | 0.1794 | _-3.544_ | -3.8295 | 0.18962676 | -0.047 | 0.3777896 | 2.066229 | 1.59020275 | 2.68475401 | | |
| 32.2 | 0.6405 | 0.3202 | _-2.966_ | -3.7825 | 0.3267517 | 0 | 0.46209668 | 2 | 1.45186098 | 2.75508472 | | |
| 32.1 | 0.389 | 0.1945 | _-3.363_ | -3.9175 | 0.2090591 | -0.135 | 0.38790769 | 2.196187 | 1.67840748 | 2.87369789 | | |
| 33.11 | 0.0322 | 0.0161 | _-4.445_ | -4.435 | 0.03648904 | -0.6525 | 0.32878279 | 3.143781 | 2.50310365 | 3.9484423 | | |
| 32.33 | 0.1384 | 0.0692 | _-4.272_ | -3.50325 | 0.23648403 | 0.2793 | 0.40335018 | 1.648038 | 1.24608258 | 2.17965535 | | |
| 32.18 | 0.4817 | 0.2408 | _-3.53_ | -3.58225 | 0.24491182 | 0.2002 | 0.40834846 | 1.7408 | 1.31166733 | 2.22709776 | | |
|  |  |  |  |  |  |  |  |  |  | |  | |
| **CNTN6** |  |  |  |  |  |  |  |  |  | | | |
| **CtR Avg** | **CtR SD** | **SEMr** | **_∆CTq_** | **∆CqTavg** | **SEM∆CT** | **∆∆CTq** | **SEM∆∆CTq** | **RCN** | **95% CI** | | | |
| 31.23 | 0.4064 | 0.2032 | _-2.894_ | -3.70475 | 0.76315461 | -0.1295 | 0.76723239 | 2.18783 | 1.28544536 | 3.72369024 | | |
| 30.93 | 0.0484 | 0.0242 | _-3.236_ | -3.37525 | 0.16670497 | 0.2 | 0.18447527 | 1.741101 | 1.53211514 | 1.97859354 | | |
| 29.94 | 0.1641 | 0.082 | _-2.112_ | -2.25525 | 0.11455861 | 1.32 | 0.1391555 | 0.80107 | 0.72741175 | 0.88218623 | | |
| 30.71 | 0.177 | 0.0885 | _-3.315_ | -3.36475 | 0.09626577 | 0.2105 | 0.12452982 | 1.728475 | 1.58553555 | 1.88430156 | | |
| 31.6 | 0.1754 | 0.0877 | _-4.2_ | -4.28775 | 0.10536446 | -0.7125 | 0.13168997 | 3.277284 | 2.99137938 | 3.59051509 | | |
| 32.09 | 0.1326 | 0.0663 | _-3.754_ | -3.5895 | 0.09162085 | -0.0143 | 0.12097503 | 2.019853 | 1.85738791 | 2.14096951 | | |
|  |  |  |  |  |  |  |  |  |  |  | | |
| **C9ORF66** |  |  |  |  |  |  |  |  |  | | | |
| **CtR Avg** | **CtR SD** | **SEMr** | **_∆CTq_** | **∆CqTavg** | **SEM∆CT** | **∆∆CTq** | **SEM∆∆CTq** | **RCN** | **95% CI** | | | |
| 31.23 | 0.4064 | 0.2032 | _-2.767_ | -3.41075 | 0.24556351 | 0 | 0.34727925 | 2 | 1.57213025 | 2.54431845 | | |
| 30.93 | 0.0484 | 0.0242 | _-3.394_ | -3.22825 | 0.0940956 | 0.1825 | 0.26297418 | 1.762349 | 1.46868507 | 2.11472994 | | |
| 29.94 | 0.1641 | 0.082 | _-2.765_ | -3.51275 | 0.53232248 | -0.102 | 0.5862326 | 2.14652 | 1.42975422 | 3.22261615 | | |
| 30.71 | 0.177 | 0.0885 | _-3.347_ | -3.42 | 0.10716424 | -0.0092 | 0.26792838 | 2.012864 | 1.67170641 | 2.42364476 | | |
| 31.6 | 0.1754 | 0.0877 | _-3.318_ | -3.518 | 0.09843259 | -0.1073 | 0.26455701 | 2.154347 | 1.79339549 | 2.58794634 | | |
| 32.32 | 0.3542 | 0.1771 | _-3.569_ | -4.01025 | 0.21749416 | -0.5995 | 0.32803224 | 3.030383 | 2.41407088 | 3.73736152 | | |
|  |  |  |  |  |  |  |  |  |  |  | | |
| **TJP2** |  |  |  |  |  |  |  |  |  | | |  |
| **CtR Avg** | **CtR SD** | **SEMr** | **_∆CTq_** | **∆CqTavg** | **SEM∆CT** | **∆∆CTq** | **SEM∆∆CTq** | **RCN** | **95% CI** | | |  |
| 30.3 | 0.2247 | 0.1124 | _-3.086_ | -2.86575 | 0.11437592 | 0.2625 | 0.14709107 | 1.667286 | 1.50567477 | 1.84624281 | | |
| 31.08 | 0.5706 | 0.2853 | _-2.367_ | -3.18825 | 0.30594147 | -0.06 | 0.31961557 | 2.084933 | 1.67062181 | 2.60199162 | | |
| 30.93 | 0.0484 | 0.0242 | _-2.994_ | -3.12825 | 0.09248746 | 0 | 0.13079702 | 2 | 1.82665349 | 2.18979682 | | |
| 29.94 | 0.1641 | 0.082 | _-3.065_ | -3.73775 | 0.46163721 | -0.6095 | 0.47081084 | 3.051462 | 2.20180952 | 4.2289847 | | |
| 30.71 | 0.177 | 0.0885 | _-2.547_ | -3.17 | 0.22684624 | -0.0418 | 0.2449758 | 2.058724 | 1.7372132 | 2.43973833 | | |
| 31.3 | 0.1591 | 0.0795 | _-3.018_ | -3.218 | 0.09122655 | -0.0898 | 0.12990848 | 2.128374 | 1.94509815 | 2.32891811 | | |
|  |  |  |  |  |  |  |  |  |  |  |  |  |
| **SMYD3** |  |  |  |  |  |  |  |  |  | | | |
| **CtR Avg** | **CtR SD** | **SEMr** | **_∆CTq_** | **∆CqTavg** | **SEM∆CT** | **∆∆CTq** | **SEM∆∆CTq** | **RCN** | **95% CI** | | | |
| 29.92 | 0.0955 | 0.0478 | _0.464_ | -0.0245 | 0.18375312 | 0.2135 | 0.22585599 | 1.724885 | 1.47492744 | 2.01720257 | | |
| 29.98 | 0.1006 | 0.0503 | _-0.319_ | -0.238 | 0.13132295 | 0 | 0.1857187 | 2 | 1.75842195 | 2.27476688 | | |
| 29.93 | 0.2391 | 0.1195 | _-0.619_ | -0.39625 | 0.13599593 | -0.1582 | 0.18905188 | 2.231865 | 1.95775194 | 2.5443581 | | |
| 31.12 | 0.2084 | 0.1042 | _-0.728_ | -0.4605 | 0.17276328 | -0.2225 | 0.21700891 | 2.333508 | 2.00762722 | 2.71228586 | | |
| 29.34 | 0.049 | 0.0245 | _0.931_ | 0.62475 | 0.12969164 | 0.8628 | 0.18456879 | 1.099807 | 0.96773313 | 1.24990526 | | |
| 30.97 | 0.0204 | 0.0102 | _-0.118_ | -0.04575 | 0.03073644 | 0.1923 | 0.13487196 | 1.750479 | 1.59424989 | 1.92201782 | | |
|  |  |  |  |  |  |  |  |  |  |  | | |
| **FH** |  |  |  |  |  |  |  |  |  | | |  |
| **CtR Avg** | **CtR SD** | **SEMr** | **_∆CTq_** | **∆CqTavg** | **SEM∆CT** | **∆∆CTq** | **SEM∆∆CTq** | **RCN** | **95% CI** | | |  |
| 33.18 | 0.3665 | 0.1832 | _-2.774_ | -3.266 | 0.18936725 | 0 | 0.26780573 | 2 | 1.66116372 | 2.40795049 | | |
| 33.31 | 0.364 | 0.182 | _-3.599_ | -3.321 | 0.1888307 | -0.055 | 0.2674266 | 2.077718 | 1.72616841 | 2.5008637 | | |
| 33.39 | 0.1541 | 0.0771 | _-3.439_ | -3.4565 | 0.14221965 | -0.1905 | 0.23682564 | 2.282317 | 1.93679823 | 2.68947568 | | |
| 34.57 | 0.3972 | 0.1986 | _-3.543_ | -3.305 | 0.20722677 | -0.039 | 0.28071852 | 2.054802 | 1.69147359 | 2.49617261 | | |
| 33.11 | 0.3109 | 0.1554 | _-3.499_ | -3.8185 | 0.15567234 | -0.5525 | 0.24514043 | 2.933248 | 2.47487968 | 3.47650882 | | |
| 33.48 | 0.0985 | 0.0492 | _-2.715_ | -2.79 | 0.05615598 | 0.476 | 0.19751822 | 1.437938 | 1.25395301 | 1.64891808 | | |
|  |  |  |  |  |  |  |  |  |  |  | | |
| **SLFN11** |  |  |  |  |  |  |  |  |  | | | |
| **CtR Avg** | **CtR SD** | **SEMr** | **_∆CTq_** | **∆CqTavg** | **SEM∆CT** | **∆∆CTq** | **SEM∆∆CTq** | **RCN** | **95% CI** | | | |
| 29.98 | 0.1006 | 0.0503 | _4.599_ | 3.571 | 0.38252313 | 0.305 | 0.42683006 | 1.618885 | 1.20427938 | 2.17622878 | | |
| 29.93 | 0.2391 | 0.1195 | _3.439_ | 3.4565 | 0.14221965 | 0.1905 | 0.23682564 | 1.752605 | 1.48727874 | 2.06526417 | | |
| 31.12 | 0.2084 | 0.1042 | _3.543_ | 3.204999 | 0.16292023 | -0.061 | 0.24980584 | 2.086378 | 0.87733212 | 1.24040106 | | |
| 29.34 | 0.049 | 0.0245 | _2.899_ | 3.468499 | 0.24537501 | 0.2025 | 0.30994976 | 1.738088 | 0.70103162 | 1.07732306 | | |
| 30.84 | 0.1566 | 0.0783 | _2.215_ | 2.515002 | 0.15549441 | -0.751 | 0.24502748 | 3.365914 | 2.84015766 | 3.98899683 | | |
|  |  |  |  |  |  |  |  |  |  |  | | |
| **LIMK1** |  |  |  |  |  |  |  |  |  | | | |
| **CtR Avg** | **CtR SD** | **SEMr** | **_∆CTq_** | **∆CqTavg** | **SEM∆CT** | **∆∆CTq** | **SEM∆∆CTq** | **RCN** | **95% CI** | | | |
| 29.24 | 0.1889 | 0.0945 | _0.485_ | 0.51775 | 0.11922551 | -0.025 | 0.17188984 | 2.034959 | 1.80639094 | 2.29244932 | | |
| 29.39 | 0.1253 | 0.0627 | _0.551_ | 0.524501 | 0.07383585 | -0.0182 | 0.14023714 | 2.025459 | 1.83784079 | 2.23223062 | | |
| 28.41 | 0.3548 | 0.1774 | _0.857_ | 0.4705 | 0.18830393 | -0.0722 | 0.20226246 | 2.10271 | 1.82764709 | 2.41917049 | | |
| 28.83 | 0.0856 | 0.0428 | _0.533_ | 0.54475 | 0.10013736 | 0.002 | 0.21327415 | 1.997229 | 1.72276498 | 2.31542046 | | |
| 28.69 | 0.1232 | 0.0616 | _0.634_ | 0.53125 | 0.06225469 | -0.0115 | 0.11791156 | 2.016006 | 1.85779151 | 2.18769517 | | |
| 28.87 | 0.1066 | 0.0533 | _0.802_ | 0.613999 | 0.23330937 | 0.0712 | 0.24147238 | 1.903627 | 1.61024268 | 2.25046463 | | |
| 32.23 | 0.2909 | 0.1454 | _0.499_ | 0.50525 | 0.16961877 | -0.0375 | 0.28845067 | 2.052668 | 1.68068508 | 2.50698049 | | |
| 28.82 | 0.1068 | 0.0534 | _0.852_ | 0.7105 | 0.06574771 | 0.1678 | 0.18191561 | 1.78046 | 1.56953165 | 2.01973406 | | |
| 31.01 | 0.1103 | 0.0551 | _0.806_ | 0.3395 | 0.38479224 | -0.2032 | 0.39036884 | 2.302578 | 1.75671614 | 3.01805431 | | |
| 28.8 | 0.1354 | 0.0677 | _0_ | -0.06625 | 0.09792505 | -0.609 | 0.39705715 | 3.050403 | 2.31649356 | 4.01682881 | | |
|  |  |  |  |  |  | \|  \| \| --- \| |  |  |  |  | | |
| **LAMB1** |  |  |  |  |  |  |  |  |  | | | |
| **CtR Avg** | **CtR SD** | **SEMr** | **_∆CTq_** | **∆CqTavg** | **SEM∆CT** | **∆∆CTq** | **SEM∆∆CTq** | **RCN** | **95% CI** | | | |
| 29.58 | 0.0721 | 0.0361 | _1.248_ | 1.156 | 0.07193871 | 0 | 0.10173669 | 2 | 1.86382099 | 2.14612885 | | |
| 29.61 | 0.1238 | 0.0619 | _1.472_ | 1.35025 | 0.09981099 | 0.1943 | 0.12303419 | 1.748054 | 1.60515791 | 1.90367026 | | |
| 28.78 | 0.0734 | 0.0367 | _1.339_ | 1.26625 | 0.09894163 | 0.1102 | 0.14054067 | 1.852855 | 1.68087138 | 2.04243572 | | |
| 29.09 | 0.189 | 0.0945 | _1.009_ | 1.281999 | 0.11170931 | 0.126 | 0.14922606 | 1.832738 | 1.65264185 | 2.03245897 | | |
| 29.3 | 0.4857 | 0.2428 | _0.153_ | 0.078751 | 0.29544484 | -1.0772 | 0.31585855 | 2.220018 | 1.69512364 | 2.62643812 | | |
| 30.49 | 0.0774 | 0.0387 | _0.924_ | 1.024 | 0.22783981 | -0.132 | 0.37309332 | 2.191623 | 0.84610351 | 1.41921456 | | |
| 28.73 | 0.1005 | 0.0503 | _0.414_ | 0.558 | 0.096181 | -0.598 | 0.24730904 | 3.027234 | 2.5503425 | 3.59329925 | | |
|  |  |  |  |  |  |  |  |  |  |  | | |
| **PTPN18** |  |  |  |  |  |  |  |  |  | | | |
| **CtR Avg** | **CtR SD** | **SEMr** | **_∆CTq_** | **∆CqTavg** | **SEM∆CT** | **∆∆CTq** | **SEM∆∆CTq** | **RCN** | **95% CI** | | | |
| 27.88 | 0.0975 | 0.0488 | _1.592_ | 1.707751 | 0.06064962 | 0.0978 | 0.21270502 | 1.868978 | 1.61277425 | 2.16588185 | | |
| 28 | 0.3885 | 0.1943 | _1.404_ | 1.61 | 0.20387508 | 0 | 0.21270502 | 2 | 1.58309816 | 2.31771796 | | |
| 27.26 | 0.0786 | 0.0393 | _1.322_ | 1.5125 | 0.05376729 | -0.0975 | 0.21084584 | 2.139835 | 1.70697721 | 2.47657393 | | |
| 28.01 | 0.2629 | 0.1314 | _1.583_ | 1.079751 | 0.16187067 | -0.5302 | 0.1705668 | 2.888358 | 2.54926907 | 3.25084867 | | |
| 26.74 | 0.7529 | 0.3764 | _2.066_ | 2.815749 | 0.43588339 | 1.2057 | 0.16823657 | 0.867088 | 0.53734432 | 1.19683167 | | |
| 27.7 | 0.0295 | 0.0148 | _1.133_ | 1.0295 | 0.05239026 | -0.5805 | 0.15891175 | 2.990734 | 2.67926697 | 3.33898912 | | |
|  |  |  |  |  |  |  |  |  |  |  | | |
| **PRTM8** |  |  |  |  |  |  |  |  |  | | | |
| **CtR Avg** | **CtR SD** | **SEMr** | **_∆CTq_** | **∆CqTavg** | **SEM∆CT** | **∆∆CTq** | **SEM∆∆CTq** | **RCN** | **95% CI** | | | |
| 29.58 | 0.0721 | 0.0361 | _-1.592_ | -1.70775 | 0.06064962 | -0.0978 | 0.21270502 | 2.140207 | 1.84682281 | 2.48019833 | | |
| 29.61 | 0.1238 | 0.0619 | _-1.404_ | -1.61 | 0.20387508 | 0 | 0.21270502 | 2 | 1.72583553 | 2.31771796 | | |
| 28.78 | 0.0734 | 0.0367 | _-1.322_ | -1.5125 | 0.05376729 | 0.0975 | 0.21084584 | 1.869303 | 1.6151345 | 2.16346832 | | |
| 29.34 | 0.4646 | 0.2323 | _-2.183_ | -2.15475 | 0.25839638 | -0.5448 | 0.26393107 | 2.917536 | 2.42976929 | 3.50321971 | | |
| 29.55 | 0.4395 | 0.2198 | _-2.066_ | -2.06575 | 0.27815378 | -0.4557 | 0.37965539 | 2.742989 | 2.10831969 | 3.56871434 | | |
| 29.74 | 0.4603 | 0.2301 | _-1.406_ | -1.6375 | 0.2513711 | -0.0275 | 0.37490927 | 2.038488 | 1.57198822 | 2.643426 | | |
| 28.95 | 0.0796 | 0.0398 | _-1.133_ | -1.2545 | 0.0424627 | 0.3555 | 0.25493237 | 1.563198 | 1.31000107 | 1.86533237 | | |
|  |  |  |  |  |  |  |  |  |  |  | | |
| **KSR1** | | | |  |  |  |  |  |  | | | |
| **CtR Avg** | **CtR SD** | **SEMr** | **_∆CTq_** | **∆CqTavg** | **SEM∆CT** | **∆∆CTq** | **SEM∆∆CTq** | **RCN** | **95% CI** | | | |
| 31.81 | 0.4102 | 0.2051 | _-1.58_ | -1.93275 | 0.23418532 | -0.1562 | 0.27609266 | 2.228773 | 1.8405759 | 2.69884583 | | |
| 32.13 | 0.2961 | 0.148 | _-1.904_ | -1.95925 | 0.22270097 | -0.1828 | 0.26642094 | 2.270091 | 1.88730722 | 2.73051137 | | |
| 31.45 | 0.0662 | 0.0331 | _-1.809_ | -1.7015 | 0.13326439 | 0.075 | 0.1978479 | 1.898684 | 1.65536814 | 2.17776443 | | |
| 32.27 | 0.5476 | 0.2738 | _-1.724_ | -1.82475 | 0.32655214 | -0.0483 | 0.3577998 | 2.068021 | 1.61378779 | 2.65010674 | | |
| 31.27 | 0.0706 | 0.0353 | _-1.701_ | -1.5225 | 0.11716626 | 0.254 | 0.18738283 | 1.677137 | 1.47285709 | 1.90974912 | | |
| 31.23 | 0.0523 | 0.0262 | _-0.515_ | -0.86625 | 0.1999509 | 0.9102 | 0.24771911 | 1.064186 | 0.44814318 | 0.63176936 | | |
| 31.31 | 0.2215 | 0.1107 | _-2.741_ | -2.47175 | 0.16929255 | -0.6952 | 0.22370597 | 3.238329 | 2.77318484 | 3.70826977 | | |
|  |  |  |  |  |  |  |  |  |  |  | | |
| **GPRC5C** | | | | |  |  |  |  |  | | | |
| **CtR Avg** | **CtR SD** | **SEMr** | **_∆CTq_** | **∆CqTavg** | **SEM∆CT** | **∆∆CTq** | **SEM∆∆CTq** | **RCN** | **95% CI** | | | |
| 28.99 | 0.363 | 0.1815 | _2.797_ | 2.81725 | 0.27386924 | 0.1643 | 0.37573232 | 1.784784 | 1.37555841 | 2.31575405 | | |
| 30.16 | 0.5651 | 0.2826 | _2.431_ | 2.4665 | 0.31663458 | -0.1865 | 0.40795572 | 2.275999 | 1.71539897 | 3.01980545 | | |
| 28.84 | 0.4008 | 0.2004 | _2.613_ | 2.6125 | 0.20310002 | -0.0405 | 0.32774995 | 2.05694 | 1.6389251 | 2.58157199 | | |
| 30.3 | 0.327 | 0.1635 | _1.855_ | 1.969751 | 0.31890295 | -0.6832 | 0.40971881 | 3.211504 | 2.41752395 | 4.26624794 | | |
| 29.25 | 0.3853 | 0.1927 | _2.451_ | 2.028501 | 0.1958677 | -0.6245 | 0.32331806 | 3.083351 | 2.46430574 | 3.85790437 | | |
| 28.74 | 0.3012 | 0.1506 | _2.742_ | 2.741 | 0.25118363 | 0.088 | 0.35953251 | 1.881652 | 1.46659209 | 2.30871411 | | |
|  |  |  |  |  |  |  |  |  |  |  | | |
| **CLSTN2** | | | | |  |  |  |  |  | | | |
| **CtR Avg** | **CtR SD** | **SEMr** | **_∆CTq_** | **∆CqTavg** | **SEM∆CT** | **∆∆CTq** | **SEM∆∆CTq** | **RCN** | **95% CI** | | | |
| 27.16 | 0.2832 | 0.1416 | _4.741_ | 4.7025 | 0.2809459 | 0.609 | 0.35304769 | 1.311302 | 1.02665546 | 1.67486809 | | |
| 27.49 | 1.1794 | 0.5897 | _4.385_ | 3.74525 | 0.60983671 | -0.3482 | 0.67143981 | 2.546031 | 1.59860123 | 4.05496573 | | |
| 26.56 | 0.0965 | 0.0482 | _4.532_ | 3.96525 | 0.29046492 | -0.1283 | 0.67547812 | 2.185934 | 1.3686675 | 3.49121266 | | |
| 27.22 | 0.3611 | 0.1805 | _3.045_ | 4.22575 | 0.54103686 | 0.1322 | 0.61407716 | 1.824815 | 1.19223862 | 2.79302275 | | |
| 28.12 | 0.3722 | 0.1861 | _3.255_ | 3.552249 | 0.24973675 | -0.5413 | 0.59589372 | 2.910467 | 1.92566532 | 4.39890495 | | |
| 27.44 | 0.311 | 0.1555 | _3.507_ | 3.86425 | 0.27256663 | -0.2292 | 0.3696769 | 2.344451 | 1.81450125 | 3.02917905 | | |
|  |  |  |  |  |  |  |  |  |  |  | | |
| **ARSB** |  |  |  |  |  |  |  |  |  | | | |
| **CtR Avg** | **CtR SD** | **SEMr** | **_∆CTq_** | **∆CqTavg** | **SEM∆CT** | **∆∆CTq** | **SEM∆∆CTq** | **RCN** | **95% CI** | | | |
| 31.49 | 0.6278 | 0.3139 | _-3.141_ | -4.1775 | 0.34633943 | -0.084 | 0.40701729 | 2.119906 | 1.59879273 | 2.81087148 | | |
| 31.24 | 0.3109 | 0.1555 | _-4.385_ | -4.17025 | 0.33951656 | -0.0768 | 0.48499742 | 2.109279 | 1.50707652 | 2.9521121 | | |
| 30.58 | 0.0572 | 0.0286 | _-4.132_ | -4.01525 | 0.05607928 | 0.0783 | 0.34411681 | 1.894412 | 1.49239876 | 2.40471627 | | |
| 31.95 | 0.534 | 0.267 | _-4.045_ | -4.72575 | 0.32231607 | -0.6322 | 0.32715827 | 3.09996 | 2.47099409 | 3.88902391 | | |
| 32.18 | 0.625 | 0.3125 | _-4.255_ | -4.05225 | 0.36371028 | 0.0413 | 0.48597615 | 1.943624 | 1.38777448 | 2.72211015 | | |
| 31.31 | 0.4477 | 0.2239 | _-3.507_ | -3.86425 | 0.27256663 | 0.2292 | 0.45450824 | 1.706157 | 1.24508291 | 2.33797302 | | |
|  |  |  |  |  |  |  |  |  |  |  | | |
| **GJA3** |  |  |  |  |  |  |  |  |  | | | |
| **CtR Avg** | **CtR SD** | **SEMr** | **_∆CTq_** | **∆CqTavg** | **SEM∆CT** | **∆∆CTq** | **SEM∆∆CTq** | **RCN** | **95% CI** | | | |
| 27.16 | 0.2832 | 0.1416 | _4.123_ | 3.920751 | 0.1456241 | 0.1705 | 0.1952069 | 1.777069 | 1.55217662 | 2.03454547 | | |
| 27.49 | 1.1794 | 0.5897 | _4.106_ | 4.0295 | 0.63174398 | 0.2792 | 0.64831076 | 1.648039 | 1.05149359 | 2.58302493 | | |
| 26.56 | 0.0965 | 0.0482 | _5.096_ | 5.004251 | 0.11689415 | 1.254 | 0.64246767 | 0.838568 | 0.53720035 | 1.30900216 | | |
| 27.22 | 0.3611 | 0.1805 | _3.406_ | 3.980999 | 0.20171023 | 0.2307 | 0.23313357 | 1.704385 | 1.45006483 | 2.0033084 | | |
| 28.37 | 0.4101 | 0.2051 | _4.44_ | 3.778749 | 0.31223766 | 0.0285 | 0.37172486 | 1.96088 | 1.51548136 | 2.53717992 | | |
| 27.69 | 0.1946 | 0.0973 | _3.06_ | 3.074 | 0.1061587 | -0.6763 | 0.32979088 | 3.195962 | 2.54287329 | 4.01678527 | | |
|  |  |  |  |  |  |  |  |  |  |  | | |
| **AKAP13** |  |  |  |  |  |  |  |  |  | | | |
| **CtR Avg** | **CtR SD** | **SEMr** | **_∆CTq_** | **∆CqTavg** | **SEM∆CT** | **∆∆CTq** | **SEM∆∆CTq** | **RCN** | **95% CI** | | | |
| 32.16 | 0.1757 | 0.0879 | _-3.587_ | -3.68875 | 0.09633167 | -0.0972 | 0.18911767 | 2.139464 | 1.87661388 | 2.43913088 | | |
| 32.09 | 0.0786 | 0.0393 | _-3.943_ | -3.77275 | 0.14413517 | -0.1812 | 0.21739515 | 2.267731 | 1.95051442 | 2.6365383 | | |
| 31.47 | 0.1544 | 0.0772 | _-3.672_ | -3.6585 | 0.11173166 | -0.067 | 0.19740736 | 2.095071 | 1.82714571 | 2.4022835 | | |
| 34.63 | 0.2453 | 0.1227 | _-3.374_ | -3.6345 | 0.12923352 | -0.043 | 0.20781483 | 2.060505 | 1.78408393 | 2.37975515 | | |
| 32.03 | 0.077 | 0.0385 | _-2.97_ | -3.453 | 0.167465 | 0.1385 | 0.23351708 | 1.816926 | 1.54540231 | 2.13615543 | | |
| 30.44 | 0.5321 | 0.266 | _-3.939_ | -3.55875 | 0.32880855 | 0.0328 | 0.36687977 | 1.955109 | 1.51610495 | 2.52123249 | | |
| 31.67 | 0.2099 | 0.1049 | _-4.299_ | -4.193 | 0.11874133 | -0.6015 | 0.2014577 | 3.034584 | 2.63909134 | 3.48934607 | | |
| 31.26 | 0.2734 | 0.1367 | _-4.302_ | -4.1085 | 0.15050781 | -0.517 | 0.22167161 | 2.86195 | 2.45432564 | 3.33727345 | | |
|  |  |  |  |  |  |  |  |  |  |  | | |
| **ABCC1** |  |  |  |  |  |  |  |  |  | | | |
| **CtR Avg** | **CtR SD** | **SEMr** | **_∆CTq_** | **∆CqTavg** | **SEM∆CT** | **∆∆CTq** | **SEM∆∆CTq** | **RCN** | **95% CI** | | | |
| 28.48 | 0.079 | 0.0395 | _3.587_ | 3.68875 | 0.09633167 | 0.0972 | 0.18911767 | 2.13149868 | 1.76082805 | | 2.50216931 | |
| 27.71 | 0.0713 | 0.0357 | _3.772_ | 3.7585 | 0.07165173 | 0.167 | 0.17781921 | 2.01505632 | 1.66653065 | | 2.36358197 | |
| 28.11 | 0.1681 | 0.084 | _3.943_ | 3.947751 | 0.09041564 | 0.3562 | 0.18617381 | 1.77759172 | 1.41269105 | | 2.14249239 | |
| 30.99 | 0.0814 | 0.0407 | _3.374_ | 3.134499 | 0.10708872 | -0.457 | 0.19481708 | 2.745372 | 2.39858672 | | 3.14229531 | |
| 28.46 | 0.1657 | 0.0828 | _2.707_ | 2.83725 | 0.22005635 | -0.7543 | 0.27369782 | 3.373511 | 2.79055712 | 4.07824607 | | |
| 28.88 | 0.2575 | 0.1288 | _2.74_ | 3.088 | 0.14303682 | -0.5035 | 0.21666849 | 2.835298 | 2.43991683 | 3.29474989 | | |
| 29.23 | 0.0566 | 0.0283 | _2.796_ | 2.9935 | 0.12748505 | -0.598 | 0.20673205 | 3.027235 | 2.62309248 | 3.35618488 | | |
| 26.88 | 0.3865 | 0.1932 | _3.939_ | 3.55875 | 0.32880855 | -0.0328 | 0.36687977 | 2.045921 | 1.58652564 | 2.63833978 | | |
